# Effectiveness of Fistuloclysis in Nutritional Management of Enteroatmospheric Fistulas: A Retrospective Study at Santo Tomás Hospital, Panama

**DOI:** 10.7759/cureus.59403

**Published:** 2024-04-30

**Authors:** Otilda M Valderrama, Martha A Quiodettis, Stephanie Monteza

**Affiliations:** 1 Trauma Unit, Hospital Santo Tomás, Panama City, PAN

**Keywords:** parenteral nutrition (pn), enteral feeding, nutritional therapy, fistuloclysis, entero-atmospheric fistula

## Abstract

Introduction

Enteroatmospheric fistulas (EAF) present significant challenges in surgical management due to their complex nature and high mortality rate. Traditional approaches often rely on prolonged parenteral nutrition, but emerging evidence suggests the potential benefits of enteral nutrition via fistuloclysis, an underappreciated enteral nutrition route. This study aims to evaluate the effectiveness of nutritional therapy, specifically fistuloclysis, in patients with EAF managed at the Trauma Unit of Santo Tomás Hospital, Panama.

Methods

A retrospective analysis was conducted on nine male patients diagnosed with EAF between January 2016 and December 2020. Data on demographics, fistula characteristics, and nutritional management were collected through chart review. Descriptive statistics were used for analysis.

Results

We analyzed nine patients, all of whom received enteral nutrition (EN) via fistuloclysis in a median of 5.5 days from the diagnosis of EAF. Seven patients required parenteral nutrition (PN) at the beginning. The use of specialized enteral formulas, supplemented with hydrolyzed proteins and medium-chain triglycerides, facilitated discontinuation of PN once 80% of nutritional requirements were met via the enteral route, and EN was continued until definitive surgery. The median duration of PN was 34 days. No adverse effects related to EN were observed, whereas complications such as central venous catheter infections were reported in all cases requiring PN.

Conclusion

Fistuloclysis is a viable and effective alternative to traditional PN in patients with EAF. Specialized nutritional strategies, including the use of semi-elemental formulas, contribute to improved outcomes and reduced complications. Early initiation and gradual increase in enteral nutrition via fistuloclysis demonstrate safety and efficacy, underscoring the importance of tailored nutritional approaches in optimizing patient care for complex surgical conditions.

## Introduction

Enteroatmospheric fistulas (EAF), a particularly complex subtype of enterocutaneous fistulas, pose significant challenges in surgical management. These complications, characterized by a high mortality rate of 42%, arise in patients lacking adequate skin coverage over the viscera, thereby creating a direct communication of enterotomy with the atmosphere [1-3]. Their treatment demands a multidisciplinary approach to ensure optimal outcomes, requiring significant commitment from the entire healthcare team. Initially, management focuses on four critical areas: controlling sepsis, correcting fluid and electrolyte imbalances, caring for the skin, and optimizing nutritional status [1,2,4,5]. 

Nutritionally, these patients often face severe depletion due to factors such as the underlying disease, nitrogen losses from the open abdomen, infections, and multiple surgeries. This condition triggers a metabolic stress response, leading to elevated energy expenditure, uncontrolled catabolism, and a reduced response to anabolic signals such as insulin, which can result in significant muscle mass loss up to 20% a week in critically ill patients. This muscle loss is a contributing factor to an increased mortality rate of 30% [6-8].

Over time, the nutritional strategy for these patients has evolved from reliance on prolonged fasting and parenteral nutrition (PN) to an emphasis on utilizing any available segment of the gastrointestinal tract for enteral nutrition (EN) [1,9]. This shift acknowledges the critical role of luminal nutrients in preventing intestinal atrophy, preserving immune function, preventing bacterial translocation, and enhancing the outcomes of anastomoses [10,11].

Fistuloclysis, as defined by Kumpf et al., involves the infusion of EN formula through the distal stoma of an enterocutaneous fistula, potentially reinfusing output from the proximal opening [1]. This method, initially suggested by Gail et al. [12] through a case where a patient transitioned from oral nutrition and PN to utilizing the distal part of the gastrointestinal tract via the fistula, has since been adopted by various groups. Despite limited high-evidence published data, emerging evidence suggests that patients undergoing this nutritional approach can significantly reduce their dependence on PN and experience fewer complications [1,9,13,14].

Specialized enteral formulas, either oligomeric or monomeric, play a pivotal role in the nutritional management of patients with EAF. These formulas are designed with partially or fully hydrolyzed nutrients, enhancing absorption without relying on gastrointestinal tract enzymes for digestion [15,16]. This characteristic makes them particularly beneficial for a substantial portion of these patients. While PN remains a crucial component of the treatment regimen, particularly during the initial phases, it is increasingly being complemented by these EN options, rather than serving as the sole nutritional intervention.

Nutritional therapy plays a critical role in achieving favorable outcomes in the reconstruction of patients with EAF. For instance, Lo et al. have demonstrated that sarcopenic patients undergoing reconstructive surgery for fistulas exhibit lower three-year survival rates, an increased risk of anastomotic leaks, complications within 30 days post operation, and a higher likelihood of dependency at discharge [17]. This highlights the crucial role of comprehensive nutritional management as a cornerstone of effective treatment strategies for these complex conditions.

In this study, we assessed the nutritional therapy used in patients with EAF managed in the trauma unit of the Hospital Santo Tomás, Panama City, Panama. Our aim was to analyze the effectiveness and outcomes of these nutritional strategies, emphasizing the integration of specialized enteral formulas alongside traditional PN methods, thereby contributing to the growing body of literature on the subject and addressing a critical gap in current knowledge.

## Materials and methods

A descriptive, longitudinal, retrospective study was carried out including all consecutive patients diagnosed with EAF managed in the trauma unit of the Hospital Santo Tomás Hospital from January 1, 2016, to December 31, 2020. We conducted a thorough chart review extracting data on demographics, fistula characteristics (location, output), and nutrition information (method, timing, duration, and tolerance). The exclusion criteria were patients who did not complete treatment at our institution.

Approach for nutrition of patients with EAF

Upon diagnosis of an enterotomy in a patient with an open abdomen, we focused on devising a comprehensive nutrition plan. The initial phase typically involved PN, simultaneously exploring avenues to establish an enteral route via fistuloclisis by the surgical team in all patients with EAF excluding distal ileal and colorectal fistulas.

Efforts to institute this enteral route usually began upon identification of an enterotomy in patients presenting with a hostile abdomen. The process generally took place in the operating room when the impossibility of resection of an intestinal segment containing an enterotomy was established. The surgeon then proceeded to evaluate the intestinal loop and placed a Foley catheter (without balloon inflation) through the enterotomies that were considered for use to nourish the patient.

The adequacy of the selected track for fistuloclysis, or the selection of an alternative enterotomy in cases where the initial choice was deemed unsuitable, was determined through a combination of surgical observations, fistulograms, and, occasionally, endoscopy conducted through the enterotomy orifice.

Following the selection of the appropriate site for nutritional administration, we started with the infusion of an oligomeric formula at a trophic rate of 20 cc/hour, with careful monitorization and incrementally increased based on patient tolerance. To meet the energy and protein requirements, we supplemented this regimen with modules of hydrolyzed proteins and medium-chain triglycerides, with the jejunum’s maximum infusion rate capped at 70cc/hour.

For the analysis, we employed descriptive statistics, presenting continuous variables as medians along with their interquartile range (IQR).

## Results

Between 2016 and 2020, the Trauma Unit managed a total of 2066 hospitalized patients, among which 10 were diagnosed with EAF. Unfortunately, the medical records of one patient could not be located, thus the analysis was conducted on the remaining nine patients.

All patients included in the study were male, aged between 16 and 52 years (mean age: 28.2 years), and presented with an open abdomen due to trauma-related injuries and developed anastomotic or small intestine suture leaks. The majority of the fistulas (n=8; 88.9%) were located in the proximal jejunum, with only one case in the distal jejunum. In one patient, the distal segment of the fistula was continuous with the colon, whereas in the others, it either terminated in another fistula or an end ileostomy. Notably, one patient had concurrent fistulae at both the duodenal level and proximal jejunum.

The nutritional therapy used is shown in Tables 1, 2. PN was initiated in 77% of the cases; however, all patients also received EN via fistuloclisis. Two patients were managed without PN, with one receiving oral nutrition in conjunction with EN from the onset due to a distal jejunum fistula, and the other managed exclusively with fistuloclysis. Reinfusion of succus entericus was not utilized.

**Table 1:** Description of patients with enteroatmospheric fistulas ^1^ Patient was discharged to perform definitive reconstruction surgery in a subsequent hospitalization; ^2^ distal segment of the fistula was continuous with the colon TPN: total parenteral nutrition; LOS: length of stay

	Anatomical site	Nutritional access used	Enteral access used	Discontinuation of TPN	Discontinuation of enteral nutrition	LOS
1	Distal jejunum	Oral and enteral	fistuloclysis	NA	Yes	38 days^1^
2	Proximal jejunum	Enteral and parenteral	fistuloclysis	Yes	no	146 days
3	Proximal jejunum^ 2^	Enteral and parenteral	fistuloclysis	Yes	no	182 days
4	Proximal jejunum	Enteral	fistuloclysis	NA	no	88 days
5	Proximal jejunum	Enteral and parenteral	fistuloclysis	Yes	no	98 days
6	Duodenum and Proximal jejunum	Enteral and parenteral	fistuloclysis	Yes	no	187 days
7	Proximal jejunum	Enteral and parenteral	fistuloclysis	Yes	no	152 days
8	Proximal jejunum	Enteral and parenteral	fistuloclysis	Yes	no	163 days
9	Proximal jejunum	Enteral and parenteral	fistuloclysis	Yes	no	146 days

**Table 2:** Characteristics of nutritional therapy used in patients with enteroatmospheric fistulas

Characteristic	n (%)
Type of Nutrition	
Oral + enteral	1 (11.1%)
Enteral + parenteral	7 (77%)
Enteral	1 (11.1%)
Enteral Nutrition Access	
Fistuloclysis	9 (100%)
Enteral Formula	
Polymeric	0 (0%)
Oligomeric + modules	9 (100%)
Fistulocisis discontinuation	1 (11.1%)
Parenteral nutrition disconstinuation	7 (100%)
Nutritional Therapy Complications	
Enteral	0 (0%)
Parenteral	7 (77.8%)
Central venous access infection	7 (77.8%)
Sepsis	1 (11.1 %)

The initiation of nutritional support post-diagnosis varied, with a median of 10 days for oral nutrition, 5.5 days for EN, and 4.5 days for PN (Table 3). It's noteworthy that, despite limited nutrient absorption orally, all patients were allowed some oral intake for psychological well-being.

**Table 3:** Central tendency measures regarding start time and duration of nutritional therapy in patients diagnosed with enteroatmospheric fistula.

	Median (Interquartile range)
Time to start enteral nutrition	5 days (4 – 7)
Time to start parenteral nutrition	3 days (2 – 4)
Time to start oral nutrition	10 days (8 – 43)
Parenteral nutrition duration	32 days (22.5 – 44.5)
Enteral Nutrition Duration	119 days (99 – 136)
Length of stay	146 days (98 – 163)

Oligomeric formulas, supplemented with hydrolyzed proteins and medium-chain triglycerides, were used in all cases through fistuloclysis. This approach enabled the discontinuation of PN once 80% of nutritional requirements were met via the enteral route, with a median PN duration of 34 days.

**Figure 1:**
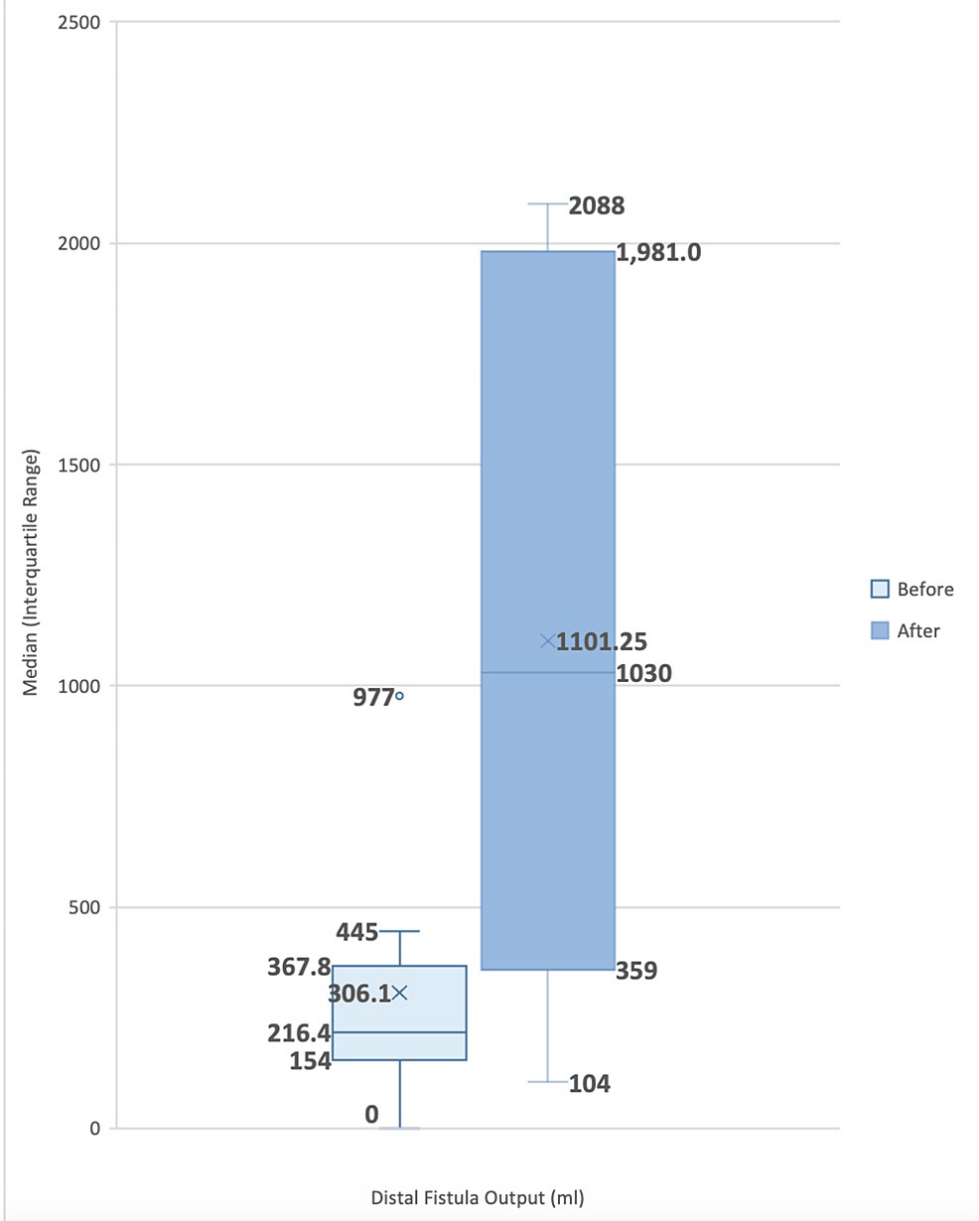
Box plot distal fistula output before and after the start of enteral nutrition in patients diagnosed with enteroatmospheric fistula

In the subset of patients where the segment used for fistuloclysis ended in another fistula or ileostomy (eight patients), there was an increase in the output from the distal opening, from an average of 306.10 ml to 1984 ml in the first week of EN (Figure 1).

The median duration of EN via fistuloclysis was 122 days, extending until the reconstructive surgery, except for one patient, with a distal jejunum fistula, who transitioned to full oral nutrition and was discharged before surgery after 26 days of fistuloclysis.

No complications were observed with EN, In contrast, all patients who required PN developed complications related to central venous catheter infections (Table 3). The median hospital stay was 149 days. All patients underwent definitive reconstructive surgery without complications.

## Discussion

Fistuloclysis, though an underutilized nutritional resource as Teubner et al. [14] suggests, has shown to be a viable option in our study, echoing the need for broader recognition and application in clinical practice. The reluctance to adopt this type of EN route, attributed by some to its complexity and aesthetic concerns for patients has been overcome in our hospital through dedicated educational efforts towards medical and nursing staff, highlighting its benefits [14,18]. This has transformed fistuloclysis from a seldom-used technique to standard practice for patients diagnosed with EAF, enabling us to achieve medium to long-term independence from PN.

The necessity to initiate mixed nutrition, combining parenteral and enteral routes, remains prevalent. However, our findings underscore the crucial role of EN in promoting intestinal adaptation and reducing the dependency on PN, which directly correlates with a reduction in complication rates [1,10,11]. This achievement necessitates meticulous planning, including contrasted and/or endoscopic studies to identify suitable intestinal segments for enteral feeding and the selection of appropriate delivery devices.

The selection of an enteral formula is critically determined by the segment of the intestine utilized for nutrition and its connection to the upper gastrointestinal tract. In our practice, similar to various studies, semi-elemental formulas are preferred for patients with EAF, primarily due to the lack of communication with the upper gastrointestinal tract which precludes access to the necessary enzymes for the digestion of complex nutrients [14,18]. Given the unavailability of elemental formulas in our country, our protocol involves the use of semi-elemental formulas for all patients which underscores the adaptability required in clinical settings, with good clinical results. Supplementation with modules of hydrolyzed proteins and medium-chain triglycerides is necessary to achieve nutritional goals. 

Our findings add to the clinical evidence of the benefit of this enteral route in the case of patients with EAF and also demonstrate a high success rate compared to other case series [9,18,19]. For example, Yin et al. implemented the use of fistuloclysis in three out of nine patients with EAF who could not establish enteral access through nasojejunal tubes [18]. Among those who used it, they were able to discontinue PN, achieving all nutrient requirements via fistuloclysis in an average of 44 days [18].

Teubner et al. further underscore the transformative potential of fistuloclysis, even among patients long-term dependent on PN [14]. In their study, 11 out of 12 patients referred to their intestinal failure center successfully replaced PN with fistuloclysis. This transition occurred within a median timeframe of 28 days from the initiation of fistuloclysis, highlighting the technique's effectiveness in facilitating nutritional autonomy.

An additional beneficial effect of fistuloclysis in the management of this type of patient is the so-called "ileal brake" that can be observed when reinfusing the succus entericus through it. It is a negative feedback mechanism in which the presence of fats in the ileum inhibits the motility of the jejunum, reducing intestinal transit, and thus improving the absorption of fluids and nutrients [1,20,21]. In our series, however, it could not be performed due to logistical challenges, but its benefit has been demonstrated by other authors [19]. Furthermore, we show that even without chyme reinfusion, fistuloclysis is a valuable option.

Safety and decrease in morbidity and mortality in the patients receiving fistuloclysis have been corroborated by various studies, including ours, which reported no adverse effects directly attributable to EN via fistuloclysis. For example, Wu et al. evaluated 95 patients, 35 of whom used fistuloclysis with re-infusion of succus entericus [13]. They reported adequate tolerance, with improvement in liver function in patients and decreased distal output. On the other hand, Yin et al. reported the survival of all patients who received this nutritional management, compared to other series published in the literature [18]. This contrasts with the complications associated with long-term PN, particularly infections related to central venous catheters.

In our series, we do not record any side effects, but Ribeiro-Junior et al. described in their systematic literature research diarrhea, vomiting, nausea, pain, abdominal distention, and one case of pneumatosis intestinalis [19]. All these side effects are usually limited and without serious complications and can be minimized using hydrolyzed macronutrients [22]. We hypothesize that the observed tolerance in our series is due to the fact that only partially or fully hydrolyzed formulas and modules were used, along with a gradual initiation and increase in their total volume and infusion rate. 

Finally, it should be noted that it is possible to allow oral intake of patients (as long as there is no obstruction), even if it does not have a nutritional objective, which will have a positive psychological effect on patients [5,11].

All these described beneficial effects of fistuloclysis have led to the American Society for Parenteral and Enteral Nutrition (ASPEN)-Federación Latinoamericana de Terapia Nutricional, Nutrición Clinica y Metabolismo (FELANPE) Guidelines recommendation that advocates for the utilization of the enteral route in patients with EAF, recommending the use of any available intestinal segment, with absorptive capacity and whose infusion site is not expected to close spontaneously, to provide nutrients by fistuloclysis, using at least a trophic dose [1].

Our study, while insightful, is not without limitations. These include a small case series without a control group and the inherent constraints of a descriptive, retrospective design, which are common in studies of this nature due to the low incidence of the complication studied. Additionally, our focus on tolerance and the description of nutrition types led to a lack of comprehensive laboratory parameters. It's important to consider these limitations when interpreting the results and conclusions of this study

## Conclusions

While enteroatmospheric fistulas remain a complex challenge, our study presents fistuloclysis as a viable, effective, and safer alternative to traditional parenteral nutrition. Specialized nutritional strategies tailored to the patients are critical. Our study highlights the potential of fistuloclysis to reduce the reliance on long-term PN, thereby mitigating the associated complications. It is a testament to the evolving landscape of nutritional management in complex surgical conditions and the benefits of adequate nutritional therapy in our patients.
